# A systematic review of digital and face-to-face cognitive behavioral therapy for depression

**DOI:** 10.1038/s41746-022-00677-8

**Published:** 2022-09-15

**Authors:** Lana Kambeitz-Ilankovic, Uma Rzayeva, Laura Völkel, Julian Wenzel, Johanna Weiske, Frank Jessen, Ulrich Reininghaus, Peter J. Uhlhaas, Mario Alvarez-Jimenez, Joseph Kambeitz

**Affiliations:** 1 Department of Psychiatry and Psychotherapy, University of Cologne, Faculty of Medicine and University Hospital of Cologne, Cologne, Germany; 2 Department of Psychiatry and Psychotherapy, Ludwig-Maximilian University, Munich, Germany; 3 Department of Public Mental Health, Central Institute of Mental Health, Medical Faculty Mannheim, University of Heidelberg, Mannheim, Germany; 4 ESRC Centre for Society and Mental Health, King’s College London, London, UK; 5 Centre for Epidemiology and Public Health, Health Service and Population Research Department, Institute of Psychiatry, Psychology and Neuroscience, King’s College London, London, UK; 6 Department of Child and Adolescent Psychiatry, Charité Universitätsmedizin, Berlin, Germany; 7 Institute of Neuroscience and Psychology, University of Glasgow, Glasgow, UK; 8 Centre for Youth Mental Health, University of Melbourne, Melbourne, VIC Australia; 9 Orygen, Parkville, VIC Australia; 10 Research Center Jülich, Institute for Cognitive Neuroscience (INM-3), Jülich, Germany

**Keywords:** Depression, Randomized controlled trials

## Abstract

Cognitive behavioral therapy (CBT) represents one of the major treatment options for depressive disorders besides pharmacological interventions. While newly developed digital CBT approaches hold important advantages due to higher accessibility, their relative effectiveness compared to traditional CBT remains unclear. We conducted a systematic literature search to identify all studies that conducted a CBT-based intervention (face-to-face or digital) in patients with major depression. Random-effects meta-analytic models of the standardized mean change using raw score standardization (SMCR) were computed. In 106 studies including *n* = 11854 patients face-to-face CBT shows superior clinical effectiveness compared to digital CBT when investigating depressive symptoms (*p* < 0.001, face-to-face CBT: SMCR = 1.97, 95%-CI: 1.74–2.13, digital CBT: SMCR = 1.20, 95%-CI: 1.08–1.32) and adherence (*p* = 0.014, face-to-face CBT: 82.4%, digital CBT: 72.9%). However, after accounting for differences between face-to-face and digital CBT studies, both approaches indicate similar effectiveness. Important variables with significant moderation effects include duration of the intervention, baseline severity, adherence and the level of human guidance in digital CBT interventions. After accounting for potential confounders our analysis indicates comparable effectiveness of face-to-face and digital CBT approaches. These findings underline the importance of moderators of clinical effects and provide a basis for the future personalization of CBT treatment in depression.

## Introduction

Cognitive behavioral therapy (CBT) is the gold-standard intervention for major depression besides pharmacotherapy^1^. Since its emergence nearly fifty years ago, a large number of studies has underlined the effectiveness of CBT in improving depressive symptoms, anxiety symptoms and psychosocial functioning^2,3^. In order to increase accessibility to CBT, recent digital CBT approaches have been developed by incorporating technological tools such as emails, smartphone apps or internet-guided therapy^4^. These approaches hold a number of potential advantages such as cost effectiveness, improved accessibility to evidence-based care for patients living in remote areas, patients living abroad or patients with immobility and - most recently - to face the challenge of providing CBT during the COVID-19 pandemic^5^.

A number of studies suggest that CBT can effectively reduce depressive symptoms, anxiety or psychosocial functioning^6–13^. In line with these promising aspects, healthcare professionals^14^ and especially young patients report to be open towards the adoption of digital treatments^15^. For patients and clinicians there is a strong preference for blended approaches which combine face-to-face CBT with digital interventions^16,17^. However, the majority of patients with depression seem to prefer face-to-face CBT^18^ and adherence to digital interventions is often low^19,20^.

Previous meta-analyses compare face-to-face with digital CBT for different conditions^21,22^ and report inconsistent results, possibly due to small samples of studies and heterogeneous interventions. Despite robust evidence for the clinical effectiveness of face-to-face and digital CBT, the equivalence of these treatments remains an open question. This represents a critical challenge for mental health professionals that need to decide which intervention should be recommended to patients and which factors should be considered when making this decision.

Our primary aim of this systematic review is to compare the effects of face-to-face vs. digital CBT interventions. The secondary aim is to investigate the moderating factors for these interventions. Overall our results indicate that after controlling for a number of potential confounders, face-to-face and digital CBT might be comparable in terms of clinical effectiveness for treating depression. We identify a number relevant factors that moderate the treatment response such as the duration of the intervention, baseline severity, adherence and the level of human guidance in digital CBT interventions.

## Results

### Literature search

**Fig. 1:**
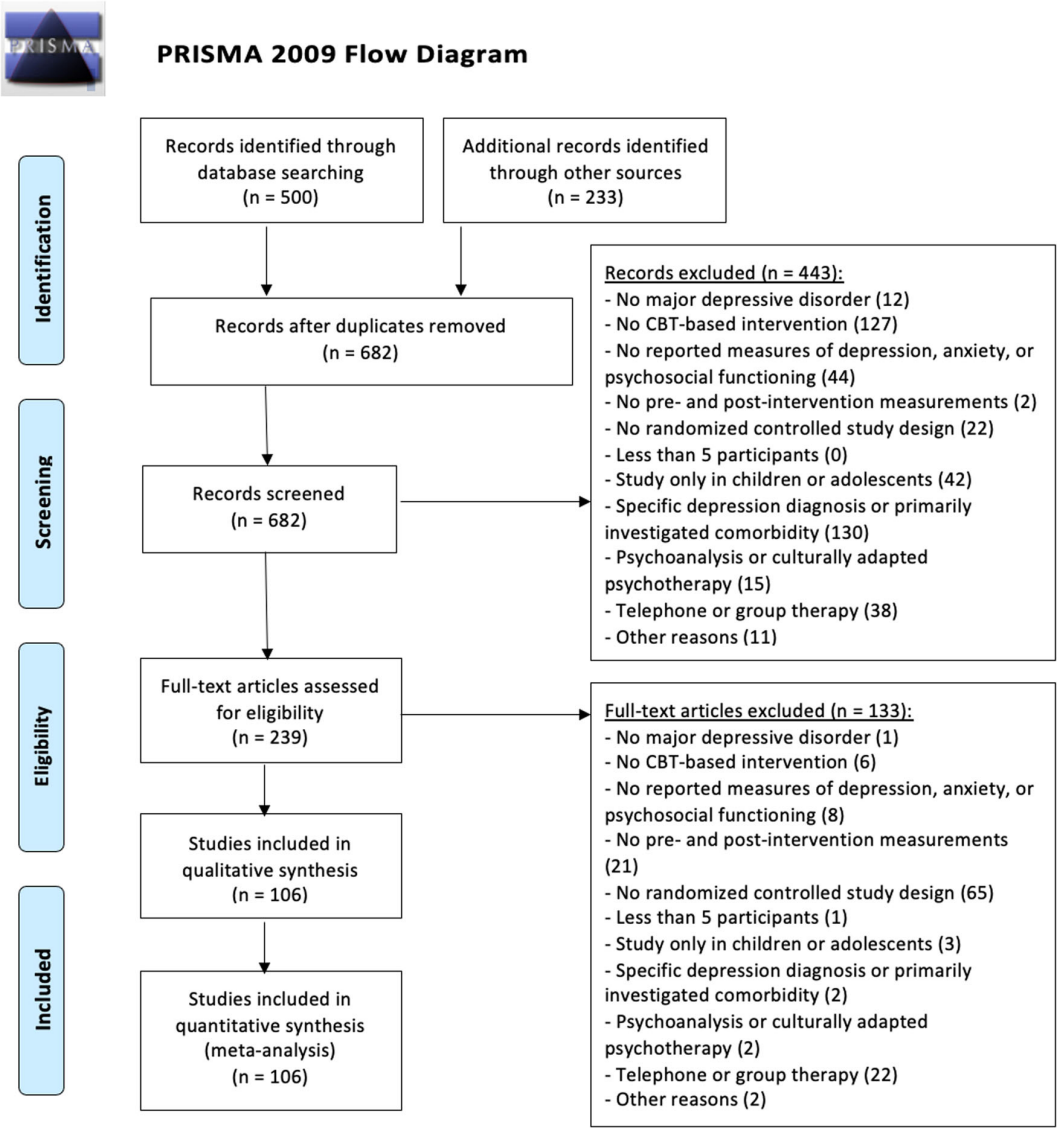
Flow-chart of the literature search according to the recommendation of the PRISMA guidelines.

We identified 682 potential studies out of which 239 studies were retrieved and assessed in full-text according to our inclusion criteria. Of the included studies, 22 face-to-face studies and 63 digital CBT studies had more than one patient sample that was eligible for inclusion due to multiple study arms. For the face-to-face CBT studies, we identified a small number of studies with a very long treatment duration (*n* = 5 studies between 1 and 6 years of treatment duration). In order to make face-to-face and digital studies more comparable, we restricted all following analyses to studies that had a treatment duration of not more than 1 year. Thus, in total *n* = 106 studies with a total of *n* = 161 samples and *n* = 11854 patients were included in the present meta-analysis (Supplementary Tables 5 and 6). This resulted in *n* = 81 samples (*n* = 3257 patients) receiving face-to-face CBT and *n* = 80 samples (*n* = 8597 patients) receiving digital CBT (see Fig. 1).

We observed significant differences between face-to-face and digital CBT samples with respect to multiple patient characteristics and other aspects of the intervention (see Table 1).

**Table 1:** Characteristics of face-to-face and digital CBT studies as included in the analysis of depressive symptoms (see supplement for an overview of included studies investigating psychosocial functioning and anxiety symptoms).

	Face-to-face studies	Digital studies	Face-to-face vs. Digital studies^a^
Number of samples	81	80	–
Mean number of patients (SD)	40.21 (40.05)	107.46 (139.83)	W = 4911.5, *p* = <0.001
Mean age (SD)	37.82 (5.47)	40.58 (5.26)	W = 3864.5, *p* = 0.001
Mean ratio of male patients	30.46%	27.09%	W = 2199.5, *p* = 0.012
Mean baseline severity (SD)^b^	31.02 (6.36)	27.29 (5.68)	W = 1634.0, *p* = 0.002
Mean ratio of patients on antidepressants	16.70%	33.93%	W = 1677.0, *p* = <0.001
Mean ratio of patients completing intervention	81.86%	72.41%	W = 1997.5, *p* = 0.001
Mean treatment duration in weeks (SD)	14.65 (8.37)	8.54 (2.89)	W = 1059.0, *p* = <0.001
Mean number of sessions (SD)	14.48 (6.26)	8.23 (3.25)	W = 1201.0, *p* = <0.001
Ratio of studies with long-term follow-up	55.56%	80.00%	X2 = 9.9, *p* = 0.002
Mean follow-up duration (months)	7.94 (6.38)	6.20 (4.69)	W = 1289, *p* = 0.322

^a^Based on two-sample Mann–Whitney-*U* test for continuous variables and on X2-test for categorical variables.

^b^Based on BDI-II scores when available or on scores converted to BDI-II with published conversion rules.

The assessment of risk of bias indicated an overall high risk of bias and comparable risk for studies investigating face-to-face CBT and studies investigating digital CBT approaches. For both interventions, the main risk of bias resulted from insufficient blinding of participants and insufficient blinding of the outcome assessment. A direct comparison indicated higher risk of selection bias (due to insufficient allocation concealment) in face-to-face CBT studies (*p* = 0.005) whereas digital CBT studies showed higher potential detection bias (blinding of outcome assessment, *p* = 0.017, Supplementary Figs. 2 and 3, Supplementary Table 4).

### Effectiveness of face-to-face vs. digital CBT

**Fig. 2:**
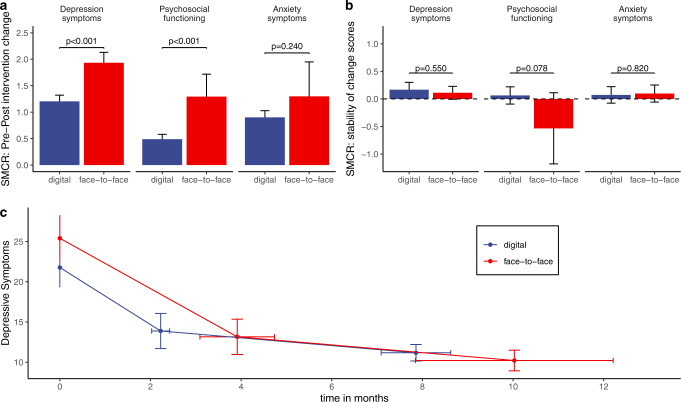
Results of meta-analyses investigating the effect of digital and face-to-face CBT interventions. **a** Effects of CBT on anxiety symptoms, depression symptoms and psychosocial functioning. **b** Results of the meta-analyses of long-term stability of treatment gains. **c** Subanalysis of samples based on depression severity based on BDI-II scores. *P* values indicate significance of differences between digital and face-to-face interventions tested by moderator analysis. Error bars indicate lower and upper limits of the 95% confidence interval. Effect sizes and *p* values are presented without correction for differences in patient samples or study design characteristics and without correction for potential publication bias.

**Fig. 3:**
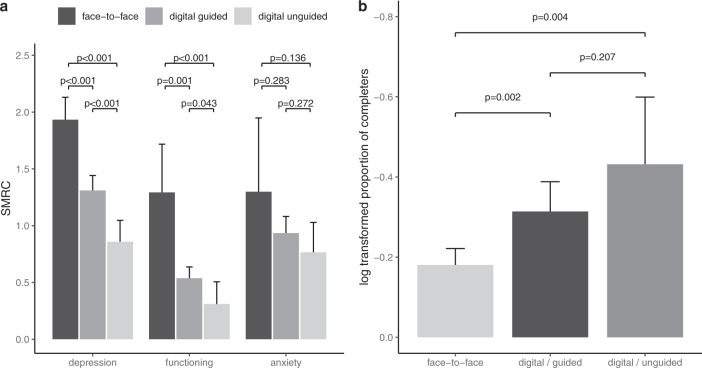
Comparison of face-to-face, guided digital and unguided digital CBT treatments regarding. **a** Clinical outcomes following the CBT intervention. **b** Comparisons of adherence. *P* values indicate significance of differences between digital and face-to-face interventions tested by moderator analysis in the meta-analytic model. Error bars indicate lower and upper limits of the 95% confidence interval.

In the analysis of depressive symptoms, face-to-face interventions (SMCR = 1.97, 95%-CI: 1.74–2.13) showed significantly stronger reductions (*p* < 0.001) as compared to digital interventions (SMCR = 1.20, 95%-CI: 1.08–1.32, Fig. 2). The difference between digital and face-to-face CBT studies remained significant after applying the trim-and-fill method to compensate for putatively missing studies (*p* < 0.001) and after controlling for differences in study design by using number of sessions and duration of intervention as covariates in the meta-analytic models (*p* = 0.010). However, there were no significant differences between digital and face-to-face CBT samples after controlling for differences in patient characteristics (mean age, gender ratio, antidepressant treatment, severity of depressive symptoms at baseline) using moderator analysis (*p* = 0.068) or when employing propensity score matching to control for differences in study design and patient characteristics (*p* = 0.700, Supplement page 5 and 6). In a subanalysis of samples based on BDI-II scores (*n* = 102 samples from 62 studies), depression scores were significantly higher in face-to-face studies as compared to digital studies at baseline (*p* = 0.048, independent *t*-test) but no differences after the intervention (*p* = 0.708, independent t-test) or at follow-up (*p* = 0.384, independent *t*-test) yielded significance (Fig. 2 and Table 1). The analysis of adherence indicated significantly fewer drop-outs in face-to-face (82.4%) as compared to digital CBT studies (72.9%, *p* = 0.014, Fig. 3, Supplement page 7 and 8). When accounting for these differences in adherence, face-to-face CBT showed stronger improvements of depressive symptoms as compared to digital CBT (*p* < 0.001).

Face-to-face studies (SMCR = 1.29, 95%-CI: 0.87–1.71) showed significantly stronger improvement in psychosocial functioning (*p* < 0.001) as compared to digital studies (SMCR = 0.49, 95%-CI: 0.39–0.58, Fig. 2). This difference remained significant after controlling for potential publication bias (*p* < 0.001) and after controlling for differences in study design by using number of sessions and duration of intervention as covariates (*p* = 0.013). However, there were no significant differences between digital and face-to-face CBT samples after controlling for differences in patient characteristics (mean age, gender ratio, antidepressant treatment, severity of depressive symptoms at baseline) using moderator analysis (*p* = 0.091) or when employing propensity score matching to control for differences in study design (*p* = 0.068, see supplement page 4 and 5).

In addition, face-to-face studies (SMCR = 1.30, 95%-CI: 0.65–1.95) showed no significant difference with regard to anxiety (*p* < 0.240) as compared to digital studies (SMCR = 0.90, 95%-CI: 0.78–1.03, see Fig. 2). These results remained unchanged when accounting for potential publication bias (*p* < 0.240). There were too few studies to conduct further analyses while controlling for additional potentially confounding variables.

All results were robust with respect to different estimates of the correlations between pre- and post-intervention assessments (*r* = 0 to *r* = 1 in steps of 0.1, Supplementary Fig. 1).

In the analysis of the long-term stability of treatment gains, face-to-face and digital interventions showed no statistical difference in depressive symptoms (*p* = 0.550), psychosocial functioning (*p* = 0.078) or anxiety symptoms (*p* = 0.820, Fig. 2, Table 1 and Supplement page 5 and 6).

### Moderator analysis

Face-to-face CBT treatments were superior to guided digital CBT treatments regarding improvement of depressive symptoms (*p* < 0.001), improvement of psychosocial functioning (*p* < 0.001) and in adherence (*p* < 0.001, see Fig. 3). At the same time, guided digital CBT was superior to unguided digital CBT regarding depressive symptoms (*p* < 0.001) and psychosocial functioning (*p* = 0.043) but there was no difference in adherence (*p* = 0.207). No differences between face-to-face CBT, guided digital CBT and unguided digital CBT were found regarding anxiety symptoms (all *p* > 0.1).

The effect of CBT on depressive symptoms was moderated by the number of sessions (*p* = 0.017) and the treatment intensity (*p* < 0.001) in face-to-face studies whereas in digital studies there was a moderation effect of the duration of the intervention (*p* = 0.034). Baseline symptom severity moderated effects of CBT on depressive symptoms in face-to-face studies (*p* = 0.038) and in digital studies (*p* = 0.029).

The effect of CBT on psychosocial functioning was moderated by age of onset of depression (*p* = 0.004) but there were too few studies to investigate this effect in digital studies. Mean age was a significant moderator in face-to-face (*p* < 0.001) but not in digital studies (*p* = 0.058). Presence of antidepressant treatment and comorbid anxiety disorder were significant moderators in face-to-face studies (*p* < 0.001 and *p* = 0.013, respectively) but not in digital studies (*p* > 0.05).

In the analysis of anxiety symptoms, the effect of CBT was moderated by the baseline severity of depressive symptoms in digital studies (*p* = 0.001) but not in face-to-face studies (*p* = 0.714).

## Discussion

Digital CBT interventions are becoming increasingly relevant for the treatment of depressive disorders. Despite the rapid proliferation of these approaches, a systematic assessment of the clinical effectiveness of CBT as compared to traditional (face-to-face) approaches, is still lacking. In the present meta-analysis we compared a total of 106 studies and over 11000 patients. To the best of our knowledge the current analysis represents the largest and most comprehensive analysis of the comparative clinical effectiveness of face-to-face and digital CBT interventions for depression. Overall, our results indicate that face-to-face approaches show superior clinical effectiveness in reducing depressive symptoms and psychosocial functioning but not in reducing comorbid anxiety symptoms. In a supplementary analysis of BDI-II equivalent scores, we largely confirmed the findings of our main analysis. Importantly, face-to-face studies were associated with higher treatment adherence. However, there were significant differences in sample-characteristics and interventions between face-to-face and digital CBT studies. Informed by knowledge that multiple factors including age, gender or disease severity at baseline may moderate treatment response (^23–26^ but see^27,28^), we employed covariate analysis and propensity score matching to control for these differences. These analyses revealed no significant differences between the face-to-face and digital interventions, suggesting that these approaches might have more comparable clinical effectiveness when accounting for moderators. Further controlled studies conducted in more comparable populations, interventions and study designs are needed to confirm these findings. Our results provide a strong foundation to initiate these efforts.

Motivated by the recent calls for precision psychiatry approaches, a number of studies have investigated potential moderators of clinical effects of face-to-face^29,30^ and digital CBT treatments^23,30,31^ with the aim to increase clinical effectiveness and to facilitate the adoption of digital tools for clinical scenarios or populations in which they are most successful.

**Fig. 4:**
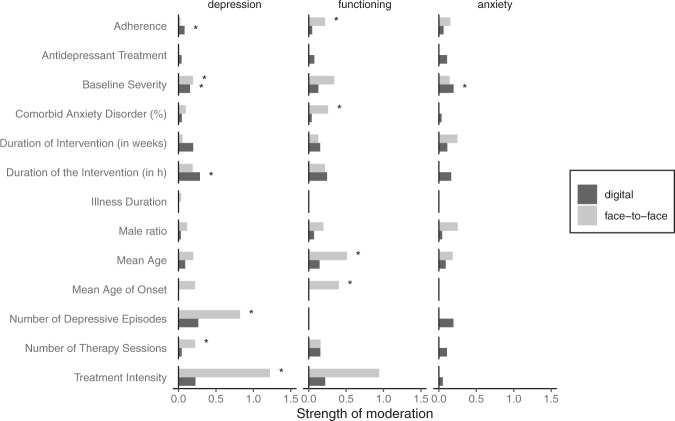
Results of the moderating analysis on depression symptoms, psychosocial functioning and anxiety symptoms. Strength of moderation was quantified by the beta-coefficient of the meta-analytic moderation model and moderation effects are plotted as absolute and sqrt values for better visualization. “*” indicates significant moderation effects (*p* < 0.05) in the meta-analytic model.

For digital CBT, some studies indicated that high baseline severity of depressive symptoms predicts improvement of depressive symptoms^24,31–35^ or psychological distress^36^. Conversely, other studies reported no such effect^28,37,38^ or even a better response to a CBT intervention delivered by trained clinicians via internet in patients with lower baseline severity of symptoms^39^. Interestingly, our findings show a significant moderation effect of baseline severity on the improvement of depressive symptoms in face-to-face CBT studies and a moderation effect of similar size in digital CBT studies (see Fig. 4). This suggests that both digital and face-to-face CBT may be suitable interventions for patients with more severe forms of depression.

In line with our findings, a recent study indicated that concurrent use of antidepressant medication is common in digital CBT trials of depression and anxiety^40^. In this analysis, digital CBT showed equivalent efficacy for patients with antidepressant medication and patients not using them^40^. Another study focused on psychological distress and found significantly higher improvements in patients on antidepressants after participating in a digital CBT programme^36^. Importantly, a high number of studies investigating face-to-face CBT, antidepressant medication was an exclusion criterion whereas this was not the case for most digital CBT studies. Thus, antidepressant medication represents a potential confound for the identified differences between digital and face-to-face CBT studies.

Treatment adherence is another important challenge for the successful implementation of digital mental health^41,42^. Previous studies investigated the role of adherence and identified adherence as a predictor of faster treatment response to digital CBT^28,35^. In the current analysis, patient characteristics and the design of the intervention were not related to adherence. However, face-to-face CBT was associated with higher adherence compared to digital CBT and no difference between guided and unguided digital CBT with respect to adherence was observed. Interestingly, our results indicate that adherence is related to the reduction of depressive symptoms in digital CBT interventions (but not in face-to-face interventions) whereas improvement of functioning was moderated by adherence in face-to-face interventions (but not in digital interventions).

In line with these findings, a higher number of sessions is an important positive predictor of the success of digital CBT treatment^39^. Interestingly, previous meta-regression analysis on the effect of the duration of CBT on treatment outcome revealed only minor effects but this analysis underlined the importance of treatment intensity (e.g. the number of treatment sessions per week)^43^.

A number of potential limitations need to be considered in the interpretation of our current findings. First, the result that face-to-face and digital CBT show similar clinical effects after the statistical correction of potential confounds remains to be confirmed in trials designed specifically to test this hypothesis. Second, we acknowledge that in the present analysis the main outcome measures are pre-post difference scores which need to be interpreted carefully as they include other effects besides the intervention such as placebo effects or the natural course of the depressive disorder. However, our main results focus on the comparison of face-to-face and digital CBT which should not lead to confounded results. Lastly, our analysis of potential biases indicated several potential risks for the majority of the included studies. This was mainly a result of insufficient blinding of participants and raters.

Face-to-face and digital CBT are effective therapy approaches for the treatment of major depression. While currently available evidence suggests robust effectiveness of face-to-face approaches, digital CBT might show comparable effects when controlling for moderators. In particular, additional human support, longer interventions and high adherence were associated with favorable treatment effects of digital CBT. Our results emphasize the potential of digital CBT to be integrated as a valuable tool in specific clinical scenarios including more severe presentations of major depression. Finally, specific moderators might guide clinicians as well as future studies in the personalization of CBT treatment for patients with depression.

## Methods

### Search strategy and selection criteria

We conducted a systematic literature search in the PubMed database to identify all relevant studies published until January 11th, 2021. In addition, primary studies in existing meta-analyses were checked for eligibility^2,7,12,22,44^. The search terms were: ((“cognitive behavioral therapy“) OR (“digital psychotherapy“ OR “psychotherapy app“ OR “mobile” OR “internet”)) AND (“major depression“) NOT (“bulimia“ OR “anorexia“ OR “psychosis” OR “bipolar“ OR “OCD“ OR “anxiety“)) NOT (“review”[Publication Type])).

We included studies that: (1) investigated patients with Major Depressive Disorder as diagnosed by the Diagnostic Statistical Manual (DSM) or International Classification of Diseases ICD, (2) employed an individual, CBT-based intervention (including second- and third-wave CBT approaches such as schema therapy, mindfulness therapy and interpersonal psychotherapy), (3) reported measures of either depressive symptoms, anxiety symptoms or psychosocial functioning (4) before and after the intervention in a (5) randomized controlled study design. We included CBT interventions administered in a face-to-face manner and CBT in a digital setting. Digital CBT could be administered in a guided or unguided manner and we included computer-based approaches (internet-based, computerized CBT-modules or email-based) as well as smartphone-based approaches.

Studies were excluded if they: (1) included less than five participants, (2) included children or adolescents (<18 years), (3) focused exclusively on a more specific depression diagnosis (i.e. postpartum depression or late-life depression), or primarily investigated somatic (e.g. HIV, diabetes) or psychiatric main diagnose preceding depressive symptomatology (e.g. panic disorder), (4) employed a psychotherapeutic intervention based on psychoanalysis or culturally-adapted psychotherapy as well as therapy delivered by a telephone or group therapy of any therapy direction.

In case some relevant data was not reported in the published manuscripts of the studies identified during the literature search, we contacted authors via email in order to obtain the missing data. In some cases we did not receive any response or the needed data was not available. Studies were excluded from our meta-analysis, if data was not sufficient to calculate effect sizes as specified in the methods section.

The procedure for this meta-analysis has been publicly registered at https://osf.io/z45xr. We follow the PRISMA reporting guidelines^45^ and additional details regarding the literature search are provided in the supplementary methods. Approval from the local ethics committee was waived as no original data was acquired in the context of this study.

### Data extraction

Depressive and anxiety symptoms were assessed by self- or observer-rated clinical scales (e.g. Beck’s Depression Inventory, Hamilton Depression Scale, State Trait Anxiety Inventory-STAI, Hamilton Anxiety Scale). In order to compare depressive symptom severity at baseline across studies, reported symptom measures were converted to BDI-II using published conversion procedures^46,47^. Psychosocial functioning was assessed using measures of global functioning (e.g. Global Assessment of Functioning), work-related functioning (e.g. Well-Being Inventory), social functioning (e.g. Social and Occupational Functioning Assessment Scale), health-related functioning (e.g. World Health Organization Quality of Life) and life quality (e.g. Quality of life scale). Adherence was quantified for all samples by the ratio of patients that did not drop out of the study and underwent an assessment after the intervention.

Literature search and data extraction were conducted independently by two researchers (L.V. and UM.R.). Discrepancies were resolved in a consensus conference (L.K.I, L.V. and UM.R.). All information was checked for potential extraction errors independently by two researchers (N.D., J.W.).

### Outcome measures

We computed the standardized mean change using raw score standardization (SMCR) describing changes between measures before and after the intervention^48^.

1 $$SMCR = \frac{{Mean_{Pre} - Mean_{Post}}}{{SD_{Pre}}}$$

Here, *Mean*_*Pre*_ and *Mean*_*Post*_ refer to the mean of clinical measures before and after the intervention and *SD*_*Pre*_ refers to the standard deviation before the intervention. As compared to the widely used standardized mean difference (SMD), SMCR accounts for the dependence of groups in pre-post study designs in the calculation of the sampling variances.

SMCRs were computed separately for the three outcome dimensions (depressive symptoms, anxiety symptoms, psychosocial functioning). In case studies reported more than one measure for a specific outcome, these measures were averaged. Long-term stability of treatment gains following CBT were analyzed by calculating changes between the post-intervention time point and the follow-up assessment. As the calculation of SMCRs requires the correlation between baseline and follow-up measures, we estimated a correlation of *r* = 0.65 based on several previous studies^49,50^. We conducted sensitivity analyses using the entire spectrum of possible correlations (0–1 with steps of 0.05) to test whether the overall effects are robust to different correlation coefficients (supplementary materials).

### Meta-analytic procedures

The main outcome was the difference in clinical effectiveness between face-to-face and digital CBT interventions. This was assessed by conducting a meta-analysis including all effect sizes (SMCR) and testing for the relevance of the factor “intervention” (face-to-face vs. digital CBT). Potential confounders including characteristics of the patient samples (mean age, gender ratio, severity of depressive symptoms at baseline, antidepressant treatment) or by differences in interventions (number of sessions, duration of intervention in weeks) was assessed by including these factors in our meta-analysis. Moreover, we investigated the moderating effect of treatment intensity which was defined as the number of CBT sessions divided by the duration of the intervention in weeks. In addition, we employed propensity score matching of face-to-face and digital CBT studies to control for differences in potentially confounding variables. In case studies did not report values for these factors, we employed median imputation. Lastly, moderator analysis was conducted to assess the role of additional factors for the clinical effectiveness of CBT interventions. Moderator analysis was conducted separately for face-to-face and digital CBT studies Table 2.

**Table 2:** Results of meta-analyses investigating pre/post effects and long-term stability of treatment gains for digital and face-to-face CBT interventions. *P* values indicate significance of meta-analytic summary effect sizes (SMCR) testing the difference between symptoms pre- vs. post-intervention. Effect sizes and *p* values are presented without correction for differences in patient samples or study design characteristics and without correction for potential publication bias.

	Face-to-face studies				Digital studies				Face-to-face vs. Digital	
Outcome	*k*	SMCR	95%-CI	*p* value	*k*	SMCR	95%-CI	*p* value	Direction	*p* value
Pre/Post analysis										
Depression symptoms	81	1.934	1.737–2.130	*p* < 0.001	80	1.204	1.086–1.321	*p* < 0.001	face-to-face > digital	*p* < 0.001
Psychosocial functioning	18	1.293	0.869–1.718	*p* < 0.001	53	0.489	0.398–0.580	*p* < 0.001	face-to-face > digital	*p* < 0.001
Anxiety symptoms	4	1.299	0.649–1.949	*p* < 0.001	44	0.902	0.775–1.029	*p* < 0.001	face-to-face > digital	*p* = 0.240
Follow-Up analysis										
Depression symptoms	45	0.112	−0.005–0.229	*p* = 0.061	64	0.167	0.031–0.302	*p* = 0.016	face-to-face < digital	*p* = 0.550
Psychosocial functioning	9	−0.533	−1.179–0.113	*p* = 0.106	45	0.064	−0.093–0.220	*p* = 0.426	face-to-face < digital	*p* = 0.078
Anxiety symptoms	3	0.098	−0.057–0.253	*p* = 0.217	36	0.073	−0.077–0.223	*p* = 0.343	face-to-face > digital	*p* = 0.820

For all meta-analyses, heterogeneity was assessed using I^2^ statistics to describe the percentage of variation across studies^51^. Higher values indicate larger heterogeneity, with I^2^ values of 25%, 50% and 75% representing low, moderate and high heterogeneity respectively^51^. Publication bias was assessed by visual inspection of funnel plots and by employing Egger’s test for funnel plot asymmetry for each meta-analysis. In case of significant Egger’s test, we used the trim-and-fill method to estimate the number of missing studies and report corrected estimated effect sizes^52^. A significance level of *p* < 0.05 (two-tailed) was used for all analyses. All reported *p* values describe summary effect sizes or moderation effects of meta-analytic models unless stated otherwise.

### Quality assessment

Two independent authors (U.M.R. and L.K.I.) assessed risk of bias using the Cochrane Risk of Bias tool^53^. We used four previously established classification criteria to quantify the risk of bias each study (high, low or unclear risk of bias): (1) random sequence generation, (2) allocation concealment, (3) selective outcome reporting (4) incomplete outcome data (5) blinding of participants and study personnel (6) blinding of outcome assessment.

### Reporting summary

Further information on research design is available in the Nature Research Reporting Summary linked to this article.

## Supplementary information

Supplemental Material

Reporting Summary

## Data Availability

All data analyzed in this meta-analysis is available upon reasonable request from the corresponding author.

## Code availability

All code for analysis is available upon reasonable request from the corresponding author. All analyses were performed using R version 4.1.1^54^ and the package metafor^55^.
