# Supplementary material for: A systematic review of digital and face-to-face cognitive behavioral therapy for depression
Source: NPJ Digit Med. 2022 Sep 15;5:144. doi: 10.1038/s41746-022-00677-8 (PMC9476400; doi:10.1038/s41746-022-00677-8)
Supplement: Supplementary file 1 — Supplemental Material [file 41746_2022_677_MOESM1_ESM.pdf]

A Systematic Review of Digital and Face-to-face Cognitive Behavioral Therapy for Depression

Lana Kambeitz-Ilankovic[1,2*]; Uma Rzayeva[1*]; Laura Völkel[1]; Julian Wenzel[1]; Johanna Weiske[2]; Frank Jessen[1]; Ulrich Reininghaus[3,4,5]; Peter J. Uhlhaas[6,7]; Mario Alvarez-Jimenez[8,9]; Joseph Kambeitz[1,10]

*these authors contributed equally

[1]Department of Psychiatry and Psychotherapy, University of Cologne, Faculty of Medicine and University Hospital of Cologne, Cologne, Germany.

[2]Department of Psychiatry and Psychotherapy, Ludwig-Maximilian University, Munich, Germany.

[3]Department of Public Mental Health, Central Institute of Mental Health, Medical Faculty Mannheim, University of Heidelberg, Mannheim, Germany

[4]ESRC Centre for Society and Mental Health, King's College London, London, UK

[5]Centre for Epidemiology and Public Health, Health Service and Population Research Department, Institute of Psychiatry, Psychology and Neuroscience, King's College London, London, UK

[6]Department of Child and Adolescent Psychiatry, Charité Universitätsmedizin, Berlin, Germany.

[7]Institute of Neuroscience and Psychology, University of Glasgow, Glasgow, U.K.

[8]Centre for Youth Mental Health, University of Melbourne, Melbourne, Victoria, Australia.

[9]Orygen, Parkville, Victoria, Australia.

[10]Research Center Jülich, Institute for Cognitive Neuroscience (INM-3), Jülich, Germany.

Differences between face-to-face and digital CBT studies:

	Face-to-face studies	Digital studies	Face-to-face vs. Digital studies[1]
Number of samples	18	53	-
Mean number of patients (SD)	46.50 (39.69)	129.68 (161.04)	W=742.5, p=<0.001
Mean age (SD)	39.15 (3.13)	41.62 (4.13)	W=608.0, p=0.031
Mean ratio of male patients	29.59 %	27.25 %	W=329.5, p=0.318
Mean baseline severity (SD)[2]	30.25 (4.77)	28.04 (4.61)	W=186.5, p=0.049
Mean ratio of patients on antidepressants	0.00 %	33.90 %	W=248.0, p=<0.001
Mean ratio of patients completing intervention	82.88 %	70.32 %	W=305.5, p=0.024
Mean treatment duration in weeks (SD)	14.46 (6.45)	8.60 (2.52)	W=138.0, p=<0.001
Mean number of sessions (SD)	13.46 (5.58)	8.35 (2.90)	W=197.5, p=0.001
Ratio of studies with long-term follow-up	50.00 %	84.91 %	X2=7.2, p=0.007
Mean follow-up duration (months)	12.78 (8.14)	6.22 (4.90)	W=99.5, p=0.010

[1]Based on two-sample Mann-Whitney-U test for continuous variables and on X2-test for categorical variables.

[2]Based on BDI-II scores when available or on scores converted to BDI-II with published conversion rules.

Supplementary Table 1: Characteristics of patients and interventions for samples in face-to-face and digital CBT studies as included in the analysis of psychosocial functioning.

	Face-to-face studies	Digital studies	Face-to-face vs. Digital studies[1]
Number of samples	4	44	-
Mean number of patients (SD)	50.50 (44.40)	104.48 (162.19)	W=113.0, p=0.360
Mean age (SD)	37.05 (2.79)	41.37 (3.97)	W=145.5, p=0.033
Mean ratio of male patients	42.15 %	26.92 %	W=12.0, p=0.005
Mean baseline severity (SD)[2]	31.34 (7.93)	26.36 (5.72)	W=55.0, p=0.347
Mean ratio of patients on antidepressants	43.33 %	34.17 %	W=48.0, p=0.876
Mean ratio of patients completing intervention	83.35 %	74.28 %	W=57.0, p=0.255
Mean treatment duration in weeks (SD)	11.75 (3.30)	8.43 (3.11)	W=36.5, p=0.066
Mean number of sessions (SD)	13.00 (4.36)	8.04 (2.93)	W=15.0, p=0.034
Ratio of studies with long-term follow-up	75.00 %	81.82 %	X2=0.0, p=1.000
Mean follow-up duration (months)	5.00 (1.73)	6.50 (5.96)	W=58.5, p=0.821

[1]Based on two-sample Mann-Whitney-U test for continuous variables and on X2-test for categorical variables.

[2]Based on BDI-II scores when available or on scores converted to BDI-II with published conversion rules.

Supplementary Table 2: Characteristics of patients and interventions for samples in face-to-face and digital CBT studies as included in the analysis of anxiety symptoms.

Differences between Face-to-face vs. Digital CBT using propensity score matching:

In order to account for the significant differences in study design and patient characteristics between digital and face-to-face CBT studies, we employed propensity score matching (PSM). In analysis of depressive symptoms, matching studies for study design differences (number of sessions, duration of the intervention) reduced the number of samples to k=42. In this subset of studies, there were no significant differences in study design and no significant differences in SMCRs between digital and face-to-face CBT studies (p=0.700). Matching studies for study design and for patient characteristics (age, gender ratio, antidepressant treatment, severity of depressive symptoms at baseline) reduced the number of samples to

k=36. In this subset of studies, there were no significant differences in study design and no significant differences in SMCRs between digital and face-to-face CBT studies (p=0.310).

In the analysis of psychosocial functioning, using PSM to account for differences in study design, reduced the number of samples to k=10. In this subset of studies, there were no significant differences in study design and no significant differences in SMCRs between digital and face-to-face CBT studies (p=0.068).

Subanalysis of differences between Face-to-face vs. Digital CBT:

In a subanalysis, we restricted face-to-face CBT studies to interventions with a more strict definition of CBT by only including studies that employed traditional forms of CBT, cognitive interventions (e.g. cognitive therapy) or behaviourally-oriented interventions (e.g. behavioral activation). In this analysis, we included n=61 samples conducting digital CBT (n=43 studies, n=6546 patients) and n=63 face-to-face CBT samples (n=48 studies, n=2195 patients). In line with the main analysis, there were significantly stronger improvements of depressive symptoms in face-to-face studies as compared to digital CBT studies (p<0.001). However, these differences did not reach significance after accounting for moderating factors such as differences in interventions and differences in patient samples (p=0.508).

In a further subanalysis, we restricted digital CBT to studies that employed a digital intervention in the form of computerized treatment modules or in the form of websites. In this analysis, we included n=72 samples employing digital CBT (n=49 studies, n=7214 patients) and n=81 face-to-face CBT samples (n=54 studies, n=3257 patients). In line with the previous findings, face-to-face CBT showed stronger improvement of depressive symptoms as compared to digital CBT (p<0.001). However, these differences did not reach significance after accounting for moderating factors such as differences in interventions and differences in patient samples (p=0.304).

Long-term stability of treatment gains following Face-to-face vs. Digital CBT:

In the analysis of the long-term stability of treatment gains, digital interventions (SMCR=0.17, 95%-CI: 0.03-0.30) showed no statistical difference (p=0.550) as compared to face-to-face interventions (SMCR=0.11 95%-CI: -0.01-0.23) for depressive symptoms. These results remained unchanged after controlling for characteristics of study design, patient sample and follow-up duration (p=0.990).

In the analysis of psychosocial functioning, face-to-face interventions showed no significant difference (p=0.078) in the stability of psychosocial functioning during the follow-up period (SMCR=-0.53, 95%-CI: -1.18-0.11) as compared to digital interventions (SMCR=0.06, 95%-CI: -0.09-0.22). Also, when controlling for study design, differences in patient characteristics and duration of follow-up period, there was no difference in the stability of treatment gains for psychosocial functioning between digital and face-to-face studies (p=0.147).

For anxiety symptoms there was no statistical difference (p=0.820) in stability of treatment gains between digital interventions (SMCR=0.07, 95%-CI: -0.08-0.22) and face-to-face interventions (SMCR=0.10, 95%-CI: -0.06-0.25). However, when controlling for study design, differences in patient characteristics and follow-up period, there was a significant difference in the stability of treatment gains (p=0.019) indicating a stronger increase in anxiety symptoms in digital as compared to face-to-face studies.

Analysis of symptom severity as measured by the Beck's depression inventory (BDI):

In a subanalysis, we restricted the extracted data to samples that reported depression symptom severity using the Beck's Depression Inventory (Version II) or similar measures (e.g. MADRS, PHQ-9, BDI Version I). All measures were transformed to the scale of the BDI-II via established procedures[1,2]. In order to avoid bias due to studies with extremely long follow-up periods, we excluded studies with a total follow-up period of more than 2 years. In this subsample, we included n=35 investigating digital CBT(k=54 samples with a total of 5569 patients with a mean age of 41.1 years) and n=31 studies of face-to-face CBT(k=48 samples with a total of 1943 patients with a mean age of 36.9 years).

There was a significant difference in the duration of the intervention ($p<0.001$) between digital CBT studies (2.23 months, SD: 0.72) and face-to-face CBT studies (3.41 months, SD: 1.44). In contrast, there was no significant difference ($p=0.446$) in the duration of the follow-up period between digital CBT studies (5.62 months, SD: 2.49) and face-to-face CBT studies (5.21 months, SD: 2.25).

Depression symptom severity at baseline was significantly lower ($p=0.048$) in patients in digital CBT studies (mean BDI score: 21.8, SD: 9.40) compared to face-to-face CBT studies (mean BDI score: 25.7, SD: 10.3). After the intervention, there was no significant difference in symptom severity ($p=0.708$) between digital (mean BDI score: 14.0, SD: 8.33) and face-to-face studies (mean BDI score: 13.3, SD: 8.01). After the follow-up period, depression symptom severity was not significantly different ($p=0.384$) in patients of face-to-face studies (mean BDI score: 10.5, SD: 3.73) as compared to patients in digital studies (mean BDI score: 11.2, SD: 3.61).

Analysis of different dimensions of psychosocial functioning:

In a subanalysis, we investigated the differences between face-to-face and digital CBT interventions in improvement of different dimensions of psychosocial functioning. Face-to-face CBT showed stronger improvement compared to digital CBT regarding general functioning ($p=0.031$), social functioning ($p=0.008$) but not regarding work functioning ($p=0.428$). There were too few studies to investigate health functioning and quality of life.

Analysis of adherence:

For the analysis of adherence (defined as the ratio of participants that completed the CBT intervention), a total of n=47 digital CBT studies (k=71 samples, 7460 patients, mean age: 40.5 years) and n=48 face-to-face CBT studies (k=67 samples, 2650 patients, mean age: 38.6 years) could be included. Across all face-to-face CBT studies adherence was 82.4 % (SD: 14.2) as compared to 72.9 % (SD: 18.9) for digital CBT studies. We conducted meta-analytic models with the log transformed proportion of adherent patients in each study

as the effect size. Differences between digital and face-to-face studies with respect to adherence were tested by employing meta-analytic models with study type (face-to-face vs. digital) as a moderator. This analysis indicated significantly higher adherence ($p<0.001$) in face-to-face CBT studies as compared to digital CBT studies. Moderator analysis indicated no significant effect of antidepressant treatment, gender ratio, mean age, number of treatment sessions, baseline severity of depression, treatment intensity of treatment spread on either adherence in face-to-face or in digital CBT studies (all $p>0.05$).

The comparison of different types of digital CBT indicated higher adherence in face-to-face compared to guided digital guided CBT ($p=0.004$) and to unguided digital PT($p=0.002$) but no significant difference between guided and unguided digital CBT ($p=0.207$).

Moderator analysis:

	Depression		Functioning		Anxiety	
**Moderator**	**Face-to-Face**	**Digital**	**Face-to-Face**	**Digital**	**Face-to-Face**	**Digital**
Male ratio	Q=1.99, p=0.159 [negative]	Q=0.01, p=0.908 [negative]	Q=3.08, p=0.079 [negative]	Q=0.64, p=0.423 [negative]	Q=1.80, p=0.180 [negative]	Q=0.04, p=0.839 [negative]
Mean Age	Q=2.69, p=0.101 [negative]	Q=0.41, p=0.522 [positive]	**Q=24.83, p<0.001 [negative]**	Q=3.60, p=0.058 [negative]	Q=0.04, p=0.841 [negative]	Q=0.24, p=0.622 [positive]
Baseline Severity	**Q=4.31, p=0.038 [positive]**	**Q=4.79, p=0.029 [positive]**	Q=3.14, p=0.077 [positive]	Q=2.39, p=0.122 [positive]	Q=0.13, p=0.714 [positive]	**Q=10.93, p=0.001 [positive]**
Antidepressant Treatment	Q=0.00, p=0.977 [negative]	Q=0.09, p=0.765 [positive]	**Q=32.17, p<0.001 [NA]**	Q=1.86, p=0.173 [positive]	-	Q=3.63, p=0.057 [positive]
Number of CBT sessions	**Q=5.69, p=0.017 [positive]**	Q=0.01, p=0.926 [negative]	Q=0.25, p=0.619 [positive]	Q=1.96, p=0.161 [positive]	-	Q=0.23, p=0.632 [negative]
Duration of intervention (weeks)	Q=0.02, p=0.889 [negative]	Q=3.79, p=0.052 [positive]	Q=0.16, p=0.686 [negative]	Q=1.65, p=0.199 [positive]	Q=0.22, p=0.641 [positive]	Q=0.34, p=0.558 [positive]
Treatment Intensity	**Q=13.34, p<0.001 [positive]**	Q=0.86, p=0.353 [negative]	Q=1.27, p=0.260 [positive]	Q=0.17, p=0.677 [negative]	-	Q=0.00, p=0.956 [negative]
Duration of the Intervention (in h)	Q=2.25, p=0.133 [positive]	**Q=4.51, p=0.034 [positive]**	Q=0.22, p=0.638 [negative]	Q=2.97, p=0.085 [positive]	-	Q=0.65, p=0.421 [positive]
Mean Age of Onset	Q=0.44, p=0.508 [positive]	-	**Q=8.30, p=0.004 [positive]**	-	-	-
Adherence	Q=0.00, p=0.948 [negative]	**Q=4.73, p=0.030 [positive]**	**Q=4.95, p=0.026 [negative]**	Q=0.85, p=0.358 [positive]	Q=1.06, p=0.304 [negative]	Q=1.17, p=0.279 [negative]
Illness Duration	Q=0.01, p=0.925 [negative]	-	-	-	-	-
Comorbid Anxiety Disorder (%)	Q=0.34, p=0.562 [negative]	Q=0.19, p=0.665 [positive]	**Q=6.13, p=0.013 [negative]**	Q=0.12, p=0.734 [negative]	-	Q=0.10, p=0.752 [positive]
Number of Depressive Episodes	**Q=14.85, p<0.001 [negative]**	Q=0.07, p=0.790 [negative]	-	-	-	Q=0.02, p=0.889 [positive]

Supplementary Table 3: Results of the moderating analysis on anxiety symptoms, depression symptoms and psychosocial functioning as reported in the main manuscript. P-values indicate significant moderation effects in the meta-analytic model. In squared brackets we indicate the direction of the moderation effects as either “positive” (higher values of moderator associated with higher improvement) or “negative” (higher values of moderator associated with lower improvement). Significant moderation effects are plotted in bold font.

Sensitivity analysis:

In order to exclude that the results reported in the present analysis are specific to the assumed correlation between pre- and post-intervention measures of r=0.65, we conducted effect size (SMRC) calculations for the entire range of possible correlations (r between 0 and 1 in steps of 0.05). These sensitivity analyses were conducted separately for face-to-face and digital CBT studies and also separately for measures of depressive symptoms, anxiety symptoms and psychosocial functioning. Overall, our results indicate that pre-post correlations only exert minor effects on estimated SMRCs and therefore unlikely bias our results.

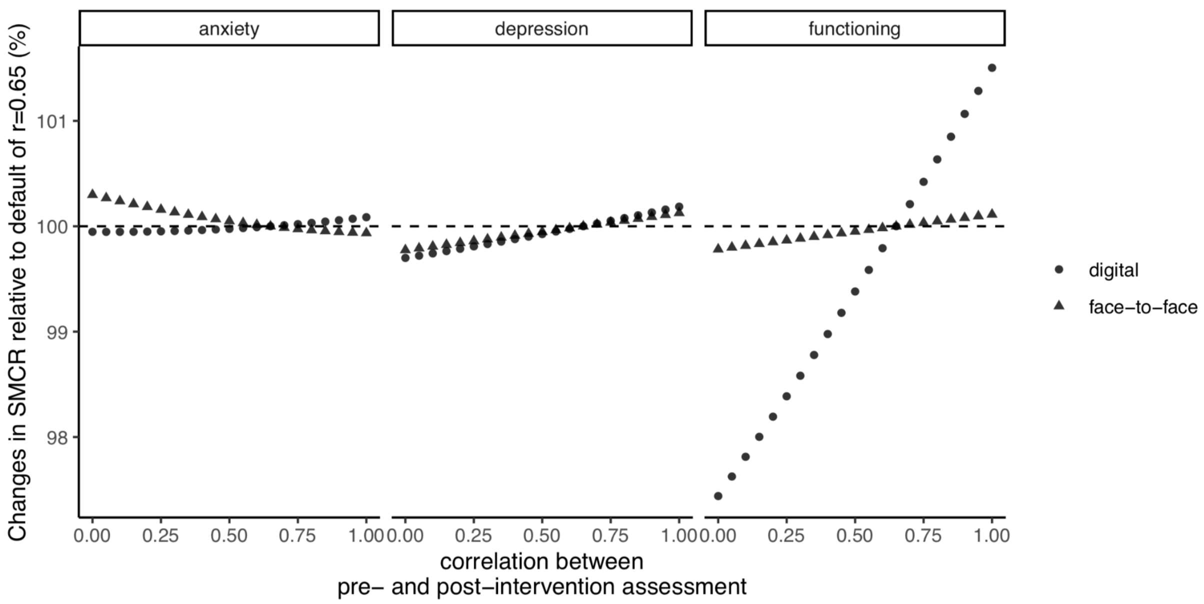

Supplementary Figure 1: Changes in effect sizes for a range of hypothetical correlations between pre- and post-intervention assessments.

Assessment of risk of bias:

Two independent authors (UM.R. and L.K.I.) assessed risk of bias using the Cochrane Risk of Bias tool[3]. We considered a trial at high risk of attrition bias if it had overall more than 50% study dropout and/or more than 30% imbalance in missing outcomes between groups. The overall risk of bias was classified as low if none of the above domains were rated as high risk and three or less are rated as unclear risk. Studies were classified as moderate if one domain was rated as high risk, or none rated as high risk but four or more rated as unclear risk. All other studies were classified as having a high risk of bias (Furukawa et al., 2016).

Assessment of risk of bias indicated an overall high risk of bias and comparable risk for studies investigating face-to-face CBT and studies investigating digital CBT approaches. For both interventions, the main risk of bias resulted from insufficient blinding of participants and insufficient blinding of the outcome assessment. A comparison between different CBT studies indicated higher risk of selection bias (due to insufficient allocation concealment) in face-to-face studies as compared to digital studies (p=0.005). There was a higher potential detection bias (blinding of outcome assessment) in digital as compared to face-to-face CBT studies (p=0.017).

Moderation analysis indicated no significant moderation effect of risk of bias resulting from allocation concealment (p=0.362), blinding of participants and personnel (p=0.893) or blinding of outcome assessment (p=0.083). Selective reporting and random sequence allocation could not be analyzed as all studies indicated low risk of bias in this domain. There was a significant moderation effect of potential risk resulting from incomplete outcome data (p<0.001). However, due to the small number of studies with high potential risk (n=1 for face-to-face studies, n=4 for digital studies), this moderation effect should be interpreted with care. Most importantly, including potential risk from incomplete outcome data, random sequence generation, blinding of participants and personnel and blinding of outcome

assessment did not effect did not change the main results and face-to-face studies still indicated significantly stronger effects (all p<0.01). Interestingly, when including potential risk from allocation concealment as a moderator in our analysis, there was no significant difference between face-to-face and digital CBT studies regarding the improvement of depression symptoms (p=0.116).

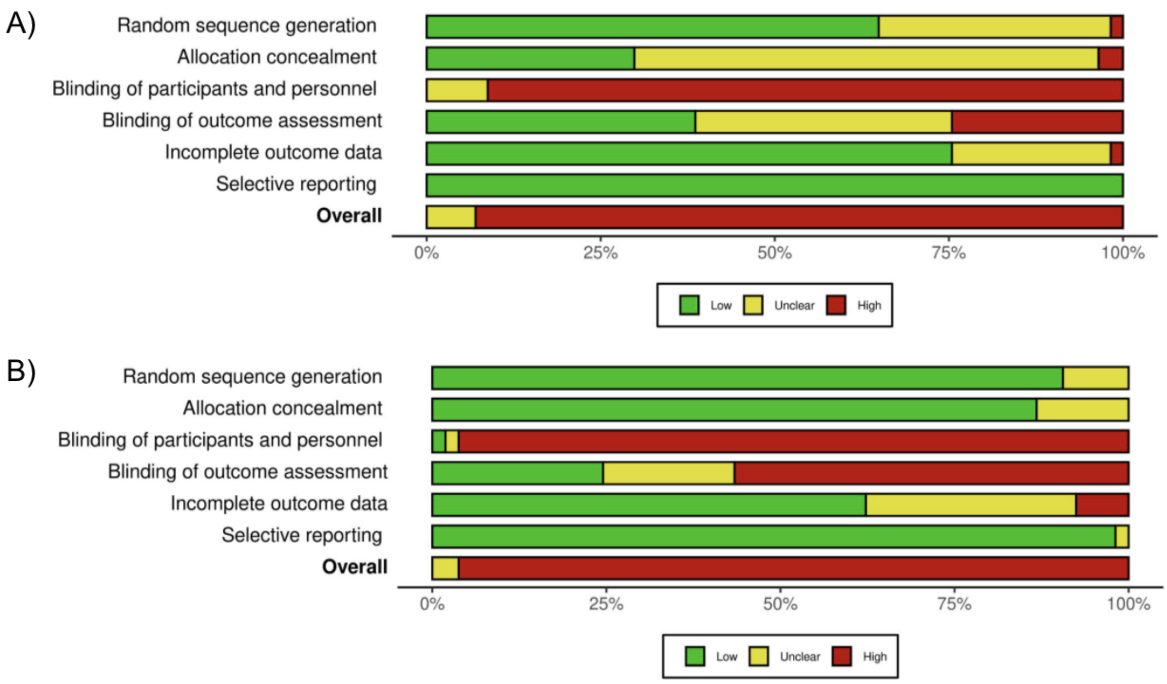

Supplementary Figure 2: Assessment of risk of bias for studies investigating face-to-face CBT (A) and for studies investigating digital CBT (B).

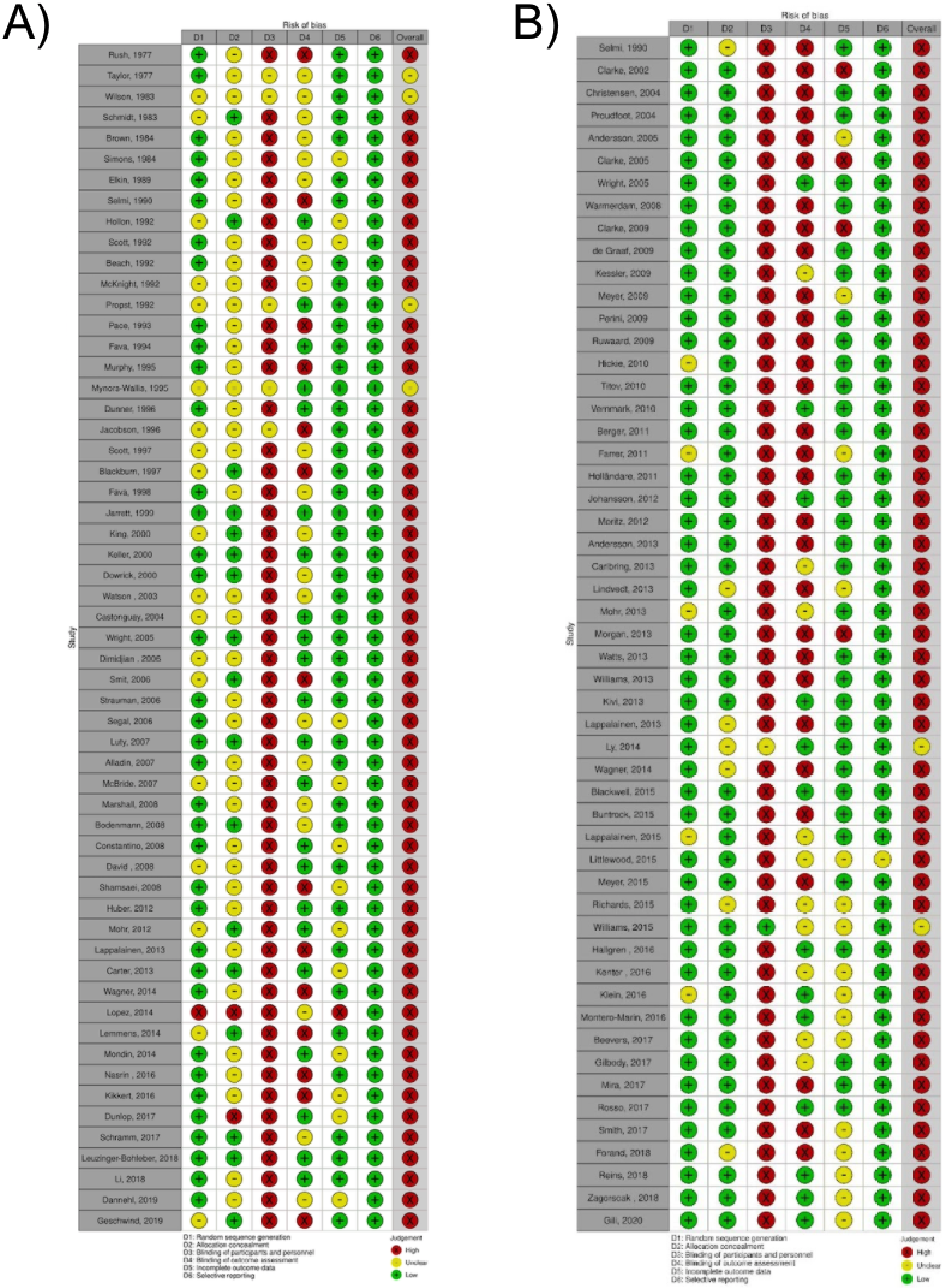

Supplementary Figure 3: Assessment of risk of bias for studies investigating face-to-face CBT (A) and for studies investigating digital CBT (B).

	face-to-face CBT	digital CBT	p-value
Random sequence generation (selection bias)	2.04 % (14.29)	0.00 % (0.00)	p=0.835
Allocation concealment (selection bias)	15.38 % (36.79)	0.00 % (0.00)	p=0.005
Blinding of participants and personnel (performance bias)	100.00 % (0.00)	96.15 % (19.36)	p=0.278
Blinding of outcome assessment (detection bias)	43.64 % (50.05)	67.19 % (47.32)	p=0.017
Incomplete outcome data (attrition bias)	1.54 % (12.40)	9.62 % (29.77)	p=0.122
Selective reporting (reporting bias)	0.00 % (0.00)	0.00 % (0.00)	NA

Supplementary Table 4: Percentage of studies with high risk of bias across different domains and comparisons between face-to-face and digital CBT studies.

Author	Year	Intervention type	n	Age (mean, sd)	Males (%)	Diagnosis	Illness duration current episode (months, mean, sd)	ADM (%)	Outcome	Therapy sessions (number)	Therapy spread (weeks)
Alladin	2007	CBT	42	35.62 (12.02)	42.8	MDD	NA	100	Anxiety Depression	NA	16
Beach	1992	CT	13	39.14 (NA)	0	MDD or dysthymia	NA	NA	Depression	17	15
Blackburn	1997	CT	27	39.6 (12)	48.1	MDD	6.8 (1.4)	0	Depression	16	16
Bodenmann	2008	CBT	19	44.35 (11.31)	35	MDD or dysthymia	NA	52.6	Depression	20	20
		IPT	18	47.33 (10.6)	40	MDD or dysthymia	NA	60	Depression	20	20
Brown	1984	CBT	13	36.5 (NA)	30	MDD	NA	30	Depression	12	8
Carter	2013	CBT	50	38.2 (12)	34	MDD	NA	0	Depression	30	52
		ST	50	38.5 (11.4)	28	MDD	NA	0	Depression	30	52
Castonguay	2004	ICT	14	38.8 (10.9)	NA	MDD	NA	NA	Depression Global Functioning	16.6	17.3
Constantino	2008	CT	7	NA	NA	MDD	NA	NA	Depression	14	15
		ICT	11	NA	NA	MDD	NA	NA	Depression	16	15
Dannehl	2019	CBT with exercise during BA	34	36.9 (10.8)	52.9	MDD	NA	41.2	Depression Health Functioning	16	16
		CBT with pleasant low-energy activities during BA	34	37.2 (12.5)	44.1	MDD	NA	38.2	Depression Health Functioning	16	16
David	2008	CT	50	39 (10)	32.1	MDD	NA	NA	Depression	20	14
		REBT	52	35 (13)	35	MDD	NA	NA	Depression	20	14

Dimidjian	2006	CT Low Severity	20	39.95 (10.28)	42.7	MDD	11 (74.9)	NA	Depression	24	16
		CT High Severity	25	39.86 (11.5)	27.5	MDD	12 (68.77)	NA	Depression	24	16
		BA Low Severity	18	39.95 (10.28)	42.7	MDD	11 (74.9)	NA	Depression	24	16
		BA High Severity	25	39.86 (11.5)	27.5	MDD	12 (68.77)	NA	Depression	24	16
Dowrick	2000	PST	89	NA	34	MDD, dysthymia, adjustment disorder	NA	NA	Depression Social Functioning	6	NA
Dunlop	2017	CBT	115	40 (11.3)	44.3	MDD	27.1 (NA)	0	Anxiety Depression	16	12
Dunner	1996	CT	10	36.7 (NA)	60	Minor depression, dysthymia	NA	0	Depression	16	16
Elkin	1989	CBT	37	35 (8.5)	NA	MDD	NA	NA	Depression Global Functioning	16	16
		IPT	47	35 (8.5)	NA	MDD	NA	NA	Depression Global Functioning	16	16
Fava	1994	CBT	20	43.7 (2.3)	40	MDD	4.6 (2.5)	100	Depression	10	20
Fava	1998	CBT	20	45.1 (10.3)	45	MDD	NA	100	Depression	10	20
Geschwind	2019	P-CBT (1. treatment block)	24	40.9 (14.7)	41.7	MDD	NA	NA	Depression Global Functioning	8	8
		P-CBT (2. treatment block)	25	40.8 (12.5)	36	MDD	NA	NA	Depression Global Functioning	8	8
		T-CBT (1. treatment block)	25	40.8 (12.5)	36	MDD	NA	NA	Depression Global Functioning	8	8
		T-CBT (2. treatment block)	24	40.9 (14.7)	41.7	MDD	NA	NA	Depression Global Functioning	8	8
Hollon	1992	CT	16	32.6	20	MDD	NA	NA	Depression	15	12

				(10.8)					Global Functioning		
Huber	2012	CBT	34	34 (6)	14.7	MDD or double depression	53.9 (72.7)	0	Depression Social Functioning	45	111
Jacobson	1996	BA	57	36.6 (NA)	28.1	MDD	NA	0	Depression	20	NA
		BA+AT	44	38.3 (NA)	22.7	MDD	NA	0	Depression	20	NA
		CT	50	39.2 (NA)	24	MDD	NA	0	Depression	20	NA
Jarrett	1999	CT	31	39.8 (1.48)	28	MDD	NA	NA	Depression Global Functioning	17	10
Keller	2000	CBASP	228	43.2 (10.8)	37.3	MDD	93.6 (120)	0	Depression	16	12
Kikkert	2016	CBT	92	38.41 (10.58)	32.6	MDD	NA	NA	Depression	16	22
King	2000	CBT	134	35 (11.4)	25.4	MDD, MDD+Anxiety	NA	0	Depression Social Functioning	5.7	NA
Lappalainen	2014	ACT	19	46.95 (12.27)	31.6	s.-r. depr. symptoms	NA	31.6	Depression	6	6
Lemmens	2014	CT	76	41.2 (12.4)	28.9	MDD	NA	0	Depression Quality of Life Work Functioning	18	28
		IPT	75	41.3 (11.8)	38.7	MDD	NA	0	Depression Quality of Life Work Functioning	18	28
Leuzinger-Bohleber	2019	CBT	41	40.43 (NA)	32.1	MDD	NA	42.1	Depression	32.5	365
Li	2018	CBT	20	27.3 (8.9)	50	MDD	NA	0	Depression	16	16
Lopez	2015	CT	83	42.8 (13.2)	15.7	MDD	NA	NA	Depression	18	18.7
Luty	2007	CBT	86	35.2 (10)	31.4	MDD	NA	0	Depression	13	16
		IPT	91	35.2 (10.5)	24.2	MDD	NA	0	Depression	13	16
Marshall	2008	CBT	37	NA	31	MDD	NA	0	Depression	16	16
		IPT	35	NA	31	MDD	NA	0	Depression	16	16

McBride	2007	CBT	21	40.71 (10.79)	31	MDD	NA	0	Depression	16	16
McKnight	1992	CT after normal DST	12	37.5 (NA)	0	MDD	NA	0	Depression	8	8
		CT after abnormal DST	10	37.5 (NA)	0	MDD	NA	0	Depression	8	8
Mohr	2012	CBT	162	47.5 (13.5)	21.6	MDD	NA	34.6	Depression	18	18
Mondin	2014	CBT	60	NA	NA	MDD	NA	NA	Depression	7	7
		CNT	60	NA	NA	MDD	NA	NA	Depression	7	7
Murphy	1995	CBT	11	39.8 (12)	27.27	MDD	NA	54.5	Depression	20	16
Mynors-Wallis	1995	PST	30	37.3 (12.8)	16.7	MDD	8.4 (NA)	0	Depression Social Functioning	6	12
Nasrin	2016	BA	20	34.9 (10.9)	35	MDD	NA	45	Depression	1	1
Pace	1993	CT	31	22.5 (NA)	19	Depr. symptoms	NA	NA	Depression	7	6
Propst	1992	CBT - NT	10	40 (NA)	17	MDD	12 (NA)	0	Depression Social Functioning	18	12
		CBT - RT	9	40 (NA)	17	MDD	12 (NA)	0	Depression Social Functioning	18	12
Rush	1977	CT	18	33.9 (NA)	31.5	Neurotic Depression	NA	NA	Anxiety Depression	15	11
Schmidt	1983	behavioral management, cognitive restructuring and assertiveness training	12	42 (NA)	16	MDD	NA	NA	Depression	7.4	8
Schramm	2017	CBASP	137	44.7 (12.1)	29.9	MDD	NA	0	Depression Global Functioning Health Functioning Quality of Life	24	20
Scott	1992	CT	29	28.8	16.7	MDD	4.2 (NA)	NA	Depression	NA	16

				(8.1)							
		BCT	18	41 (10.4)	NA	MDD	8.6 (5.4)	NA	Depression	6	6
Scott	1997	BCT	18	41 (10.4)	NA	MDD	8.6 (5.4)	NA	Depression	6	6
Segal	2006	CBT	149	37.89 (11.25)	44.3	MDD	7.8 (5.8)	41.7	Depression	20	23
Selmi	1990	CBT	12	24.7 (4.33)	33.3	MDD or minor depression	NA	NA	Depression	6	6
Shamsaei	2008	CT	40	36 (11)	10	MDD	NA	0	Depression	8	8
Simons	1984	CBT	14	31 (NA)	14.3	MDD	NA	0	Depression	20	12
Smit	2006	CBT	36	42.8 (11.6)	46	MDD	NA	42	Depression	11	11
Strauman	2006	CT	18	40.1 (16.3)	38.9	MDD or dysthymia	NA	NA	Depression	19	NA
		SST	21	37.2 (13.3)	14.3	MDD or dysthymia	NA	NA	Depression	21	NA
Taylor	1977	CT	7	22.4 (2.6)	28.6	MDD	NA	0	Depression	6	NA
		BT	7	22.4 (2.6)	28.6	MDD	NA	0	Depression	6	NA
		CBT	7	22.4 (2.6)	28.6	MDD	NA	0	Depression	6	NA
Wagner	2014	CBT	30	38.7 (11.4)	50	MDD	NA	30	Anxiety Depression	8	8
Watson	2003	CBT	33	41.52 (10.82)	33	MDD	NA	0	Depression Social Functioning	16	16
Wilson	1983	BT	7	39.5 (NA)	25	MDD	NA	NA	Depression	8	8
		CT	5	39.5 (NA)	12.5	MDD	NA	NA	Depression	8	8
Wright	2005	CT	15	41.9 (9)	26.7	MDD	NA	0	Depression	9	8

ACT = acceptance and commitment therapy
A-CT = acute-phase cognitive therapy
ADM = antidepressant medication
AT = automatic thoughts
BA = behavioral activation
BCT = brief cognitive therapy
BT = behavioral therapy
CBASP = cognitive behavioral analysis system of psychotherapy
CBT = cognitive behavioral therapy
CBT - NT = CBT with non-religious therapist
CBT - RT = CBT with religious therapist
CNT = cognitive narrative therapy
CT = cognitive therapy
depr. = depressive
DST = dexamethasone suppression test
ICT = integrative cognitive therapy
IPT = interpersonal therapy

P-CBT = positive CBT
PST = problem solving therapy
REBT = rational emotive behavior therapy
ST = schema therapy
T-CBT = traditional CBT

Supplementary Table 5: Overview of studies investigating face-to-face CBT for the treatment of depression.

Author	Year	Intervention type	Deliverance	Guidance	Guidance type	Software	n	Age (mean, sd)	Males (%)	Diagnosis	Illness duration (months)	ADM (%)	Outcome	Therapy sessions	Therapy spread (weeks)
Andersson	2005	eCBT	CM	yes	email	JSP and MySQL	36	36.4 (11.5)	22	MDD	NA	22	Depression Anxiety Quality of Life	5	10
Andersson	2013	eCBT	CM	yes	email	NA	33	42.8 (14.9)	24.2	MDD	NA	27.2	Depression Anxiety Quality of Life	8	NA
Beevers	2017	eCBT	CM	yes	telephone and email	Deprexis	285	31.2 (10.7)	25.6	MDD	NA	39.6	Depression Global Functioning	11	8
		eCBT	CM	yes	telephone and email	Deprexis	91	34.1 (12.4)	22	MDD	NA	48.3	Depression	11	8
Berger	2011	eCBT	CM	no	NA	Deprexis	25	38.6 (14.2)	28	MDD or dysthymia	NA	NA	Depression Quality of Life Social Functioning	10	10
		eCBT	CM	yes	email	Deprexis	25	38.2 (15.1)	32	MDD or dysthymia	NA	NA	Depression Quality of Life Social Functioning	10	10
Blackwell	2015	positive CBM	website	yes	telephone and email	NA	76	37.64 (14.1)	32	MDD	NA	43	Depression Anxiety Quality of Life	12	4
Buntrock	2015	eCBT + ePST	CM	yes	messages	GET.ON Mood Enhancer	202	45.7 (11.93)	26	subthreshold depression	NA	24.8	Depression Anxiety Health Functioning	6	4.5
		OPE	information website	no	NA	NA	204	44.38 (11.84)	26	subthreshold depressi	NA	21.6	Depression Anxiety Health Functioni	NA	NA

										on			ng		
Carlbri ng	2013	eBA + ACT	CM	yes	email	Depress ionshjäl pen	40	43.6 (13.7)	22.5	MD D	NA	10	Depressi on Anxiety Quality of Life	7	8
Christe nsen	2004	eCBT	CM	yes	technic al teleph one suppor t	MoodG ym	18 2	35.85 (9.5)	25	dep r. sym pto ms	NA	NA	Depressi on	5	6
		OPE	infor matio n websi te	yes	technic al teleph one suppor t	BluePag es	16 5	37.25 (9.4)	30	dep r. sym pto ms	NA	NA	Depressi on	5	6
Clarke	2002	eCBT	CM	no	NA	ODIN	14 4	43.3 (12.2)	26.4	s.-r. dep r. sym pto ms	NA	NA	Depressi on	2.6	NA
Clarke	2005	eCBT	CM	no	postcar d remind ers	ODIN	75	50.3 (10.8)	28	s.-r. dep r. sym pto ms	NA	NA	Depressi on Health Functioni ng	5.9	NA
		eCBT	CM	yes	teleph one	ODIN	80	44.4 (10.5)	16.2	s.-r. dep r. sym pto ms	NA	NA	Depressi on Health Functioni ng	5.6	NA
Clarke	2009	eCBT	CM	yes	postcar d and teleph one remind ers	ODIN	83	22.6 (2.3)	19	s.-r. dep r. sym pto ms	NA	NA	Depressi on	8.5	NA
de Graaf	2009	eCBT	CM	no	NA	Colour Your Life	10 0	44.3 (11.8)	48	MD D	NA	0	Depressi on Social Functioni ng Work Functioni ng	9	9
		eCBT	CM	yes	GP consult ation	Colour Your Life	10 0	45.2 (10.9)	37	MD D	NA	NA	Depressi on Social Functioni ng Work Functioni ng	9	9

Farrer	2011	OPE + eCBT	CM + information website	no	NA	BluePages + MoodGYM	38	37.5 (12)	14	depr. symptoms	NA	NA	Depression	6	6
		OPE + eCBT	CM + information website	yes	technical telephone support	BluePages + MoodGYM	45	41.7 (12.1)	18	depr. symptoms	NA	NA	Depression	6	6
Forand	2018	eCBT	CM	yes	telephone and email	Beating the Blues	60	33.3 (12.9)	26.7	MDD	NA	44.1	Depression	8	8
		eCBT	CM	yes	telephone and email	Beating the Blues	20	32.4 (10.6)	NA	MDD	NA	26.7	Depression	8	8
Gilbody	2017	eCBT	CM	yes	technical telephone support	MoodGYM	182	40.3 (13.7)	37.9	MDD	NA	39	Depression Anxiety Health Functioning	6	6
		eCBT	CM	yes	telephone	MoodGYM	187	41 (13.8)	33.2	MDD	NA	38.5	Depression Anxiety Health Functioning	6	14
Gili	2020	HLP	CM	yes	telephone, SMS and email + one face-to-face group session	NA	54	44.67 (9.98)	25.9	MDD or dysthymia	NA	NA	Depression Health Functioning	4	6
		MT	CM	yes	telephone, SMS and email + one face-to-face group session	NA	54	47.5 (13.09)	13	MDD or dysthymia	NA	NA	Depression Health Functioning	4	6
		PAPP	CM	yes	telephone, SMS and email + one face-to-face group	NA	56	44.53 (10.23)	21.4	MDD or dysthymia	NA	NA	Depression Health Functioning	4	6

					session										
Hallgren	2016	eCBT	CM	yes	telephone and email	NA	317	43 (12)	27.8	MDD	NA	31	Depression	13	12
Hickie	2010	eCBT	CM	yes	GP consultation	MoodGYM	29	33.7 (NA)	30	MDD	NA	0	Global Functioning	NA	8
Holländare	2011	eCBT	CM	yes	email	NA	42	44.8 (13.9)	14.3	MDD	NA	42.8	Depression Anxiety Quality of Life	16	10
		non-specific therapy	emails	yes	email	NA	42	45.8 (11.9)	16.7	MDD	NA	47.1	Depression Anxiety Quality of Life	NA	10
Johansson	2012	tailored eCBT	CM	yes	email	NA	39	45.7 (10.9)	25.6	MDD	NA	23.1	Depression Anxiety Quality of Life	9.7	10
		standardized eCBT	CM	yes	email	NA	40	43.7 (13.7)	30	MDD	NA	30	Depression Anxiety Quality of Life	8	10
Kenter	2016	ePST	CM	yes	email	NA	136	38.6 (10.5)	42.6	MDD	NA	21.3	Depression Anxiety Quality of Life	5	5
Kessler	2009	eCBT	online chat	yes	messages	NA	149	35.6 (11.9)	30.9	MDD	NA	54	Depression Quality of Life	10	16
Kivi	2014	eCBT	CM	yes	telephone and email	Depressionshjälpen	45	36.6 (11.3)	34	MDD	NA	26.7	Depression Anxiety	7	12
Klein	2016	eCBT	CM	yes	email	Deprexis	509	42.8 (11)	31.2	depr. symptoms	NA	48.9	Depression Health Functioning	11	12
Lappalainen	2015	iACT	CM	yes	messages and email	Good Life Compass	19	50.32 (12.54)	31.6	s.-r. depr. symptoms	73 (64.6)	20.5	Depression	6	7
Lappalainen	2014	iACT	CM	yes	messages and email	Good Life Compass	19	42.26 (16.04)	31.6	s.-r. depr. sym	NA	36.8	Depression	6	6

										ptoms					
Lindvedt	2013	eCBT	CM + information website	no	NA	MoodGYM + Bluepages	81	28.8 (7.2)	32.1	depr. symptoms	NA	NA	Depression	5	8
Littlewood	2015	eCBT	CM	yes	telephone	Beating the Blues	210	39.6 (12.3)	32.4	MDD	NA	NA	Depression Social Functioning Health Functioning	8	NA
		eCBT	CM	yes	telephone	MoodGYM	242	39.4 (13)	35.1	MDD	NA	NA	Depression Social Functioning Health Functioning	6	NA
Ly	2014	eBA	mobile app	yes	SMS and email	NA	40	36.6 (10.5)	30	MDD	NA	30	Depression Anxiety Quality of Life	NA	8
		MT	mobile app	yes	email	NA	41	35.6 (11.3)	29.3	MDD	NA	34.1	Depression Anxiety Quality of Life	NA	8
Meyer	2009	eCBT	CM	no	NA	Deprexis	320	34.58 (11.53)	23	s.-r. depr. symptoms	NA	43	Depression Work Functioning	12	9
		eCBT	CM	no	NA	Deprexis	57	35.25 (11.79)	30	s.-r. depr. symptoms	NA	52	Depression Work Functioning	12	9
Meyer	2015	eCBT	CM	no	NA	Deprexis	78	44 (11.02)	25.6	MDD	NA	50	Depression Anxiety Health Functioning	NA	12
Mira	2017	eCBT	CM	no	NA	Sonreíres Divertido	36	35.22 (9.7)	36.1	depr. symptoms	NA	NA	Depression Anxiety	8	12
		eCBT	CM	yes	teleph	Sonreír	44	35.05	34.1	dep	NA	NA	Depressi	8	12

					one	es Divertid o		(9.36)		r. sym pto ms			on Anxiety		
Mohr	2013	eCBT	CM	yes	teleph one and email	moodM anager + TeleCoa ch	34	47.6 (12.4)	26.5	MD D	NA	35.3	Depressi on	18	12
		eCBT	CM	no	NA	moodM anager	35	48.9 (14.6)	28.6	MD D	NA	34.3	Depressi on	18	12
Monte ro-Mar in	2016	eCBT	CM	no	NA	Smiling is fun	98	42.57 (11.94)	26.5	MD D	NA	NA	Depressi on Quality of Life Health Functioni ng	10	12
		eCBT	CM	yes	email	Smiling is fun	96	43.19 (9.3)	20.8	MD D	NA	NA	Depressi on Quality of Life Health Functioni ng	10	12
Morga n	2013	self-he lp strateg ies	auto mate d email s	no	NA	Mood Memos	86 2	37.7 (13.7)	20.6	dep r. sym pto ms	NA	NA	Depressi on Anxiety Work Functioni ng	12	6
Moritz	2012	eCBT	CM	no	NA	Deprexi s	10 5	38 (10.76)	22.9	dep r. sym pto ms	NA	21	Depressi on Quality of Life	12	8
Perini	2009	eCBT	CM	yes	email, discuss ion group moder ation	Sadness progra mme	27	49.28 (9.38)	14.8	MD D	NA	40.7 4	Depressi on Anxiety Global Functioni ng	6	8
Proudf oot	2004	eCBT	CM	yes	technic al suppor t	Beating the Blues	14 6	42.6 (14.3)	27	MD D or anxi ety	NA	44.5	Depressi on Anxiety Work Functioni ng	8	8
Reins	2018	eCBT	CM	yes	messa ges	GET.ON Mood Enhanc er	65	40.6 (10.7)	30.8	MD D	NA	NA	Depressi on Anxiety Health Functioni ng	6	6
		OPE	CM	no	NA	GET.ON Mood Enhanc er	66	42.7 (10.8)	18.2	MD D	NA	NA	Depressi on Anxiety Health	NA	NA

													Functioning		
Richards	2015	eCBT	CM	yes	messages	Space from Depression	96	40.63 (11.17)	26	depr. symptoms	NA	19.8	Depression Anxiety Work Functioning	7	8
Rosso	2017	eCBT	CM	yes	telephone	Sadness Program	37	29.2 (7.69)	37.8	MDD	NA	NA	Depression Anxiety	6	10
Ruwaard	2009	eCBT	CM	yes	email	NA	36	42 (10)	25	MDD	NA	20	Depression Anxiety	8	16
Selmi	1990	eCBT	CM	no	NA	NA	12	28.9 (4.89)	41.7	MDD or minor depression	NA	NA	Depression	6	6
Smith	2017	eCBT	CM	yes	telephone and email	Sadness Program	54	42.5 (12.63)	14.8	MDD	NA	44.4	Depression Anxiety	6	12
Titov	2010	eCBT	CM	yes	technical support	Sadness Program	41	44 (12.28)	36.5	MDD	NA	NA	Depression Anxiety Global Functioning	6	8
		eCBT	CM	yes	telephone or email, discussion group moderation	Sadness Program	46	40 (12.33)	13	MDD	NA	NA	Depression Anxiety Global Functioning	6	8
Vernmark	2010	eCBT	emails	yes	email	NA	30	40.5 (13.9)	30	MDD	NA	NA	Depression Anxiety Quality of Life	8	8
		eCBT	CM	yes	email	NA	29	37.2 (13)	20.7	MDD	NA	NA	Depression Anxiety Quality of Life	7	8
Wagner	2014	eCBT	CM	yes	messages	NA	32	37.3 (11.4)	22	MDD	NA	9	Depression Anxiety	16	8
Warmerdam	2008	eCBT	CM	yes	email	based on “Coping with Depress	88	45.7 (NA)	30.1	MDD	NA	NA	Depression Anxiety Quality of Life	8	8

						ion”									
		eCBT	CM	yes	email	NA	88	45.1 (NA)	35.2	MDD	NA	NA	Depression Anxiety Quality of Life	5	5
Watts	2013	eCBT	mobile app	yes	telephone and email	Sadness Program	15	41 (12.38)	20	MDD	NA	NA	Depression Anxiety	6	8
		eCBT	CM	yes	telephone and email	Sadness Program	20	41 (12.38)	20	MDD	NA	NA	Depression Anxiety	6	8
Williams	2013	CBM-I	CM	no	NA	NA	38	44.28 (11.78)	23	MDD	NA	49	Depression Anxiety	7	1
		CBM-I + eCBT	CM	yes	email	Sadness Program	38	44.28 (11.78)	23	MDD	NA	49	Depression Anxiety Global Functioning	13	11
Williams	2015	pos. CBM-I	CM	no	NA	NA	36	43.94 (10.8)	31	MDD	NA	50	Depression Anxiety	7	1
		pos. CBM-I + eCBT	CM	yes	telephone and email	Sadness Program	36	43.94 (10.8)	31	MDD	NA	50	Depression Anxiety Global Functioning	13	11
		pos. + neg. CBM-I + eCBT	CM	yes	telephone and email	Sadness Program	39	39.86 (11.93)	23	MDD	NA	54	Depression Anxiety Global Functioning	13	11
Wright	2005	eCT	CM	yes	therapy session	NA	15	38.2 (9.8)	26.7	MDD	NA	0	Depression	8	8
Zagorscak	2018	eCBT	CM	yes	individual messages	NA	555	45.7 (11.8)	35.1	affective disorder	NA	32.3	Depression Anxiety Social Functioning	7	6
		eCBT	CM	yes	standard messages	NA	534	45.8 (10.7)	33.7	affective disorder	NA	31.3	Depression Anxiety Social Functioning	7	6

ACT = acceptance and commitment therapy
ADM = antidepressant medication
CBM (-I) = (imagery) cognitive bias modification
CM = computerized modules

depr. = depressive
eBA = electronic behavioral activation
eCBT = electronic cognitive behavioral therapy
ePST = electronic problem solving therapy
GP = general practitioner
HLP = healthy lifestyle psychoeducational program
IPT= interpersonal therapy
MDD = major depressive disease
MT = mindfulness therapy
neg. = negative
OPE = online psychoeducation
PAPP = positive affect promotion program
pos. = positive
PP = positive psychology
s.-r. = self-reported

Supplementary Table 6: Overview of studies investigating digital CBT for the treatment of depression.

Supplementary References:

1. Hawley, C. J. *et al.* Equations for converting scores between depression scales (MÅDRS, SRS, PHQ-9 and BDI-II): good statistical, but weak idiographic, validity. *Hum. Psychopharmacol.* **28**, 544–551 (2013).
2. Furukawa, T. A. *et al.* Translating the BDI and BDI-II into the HAMD and vice versa with equipercentile linking. *Epidemiol. Psychiatr. Sci.* **29**, e24 (2019).
3. Higgins, J. P. T. *et al.* The Cochrane Collaboration's tool for assessing risk of bias in randomised trials. *BMJ* **343**, d5928 (2011).
