# Supplementary material for: A systematic review of digital and face-to-face cognitive behavioral therapy for depression
Source: NPJ Digit Med. 2022 Sep 15;5:144. doi: 10.1038/s41746-022-00677-8 (PMC9476400; doi:10.1038/s41746-022-00677-8)
Supplement: Supplementary file 2 — Reporting Summary [file 41746_2022_677_MOESM2_ESM.pdf]

nature portfolio

Corresponding author(s): Joseph Kambeitz

Last updated by author(s): 2022 08 03

Reporting Summary

Nature Portfolio wishes to improve the reproducibility of the work that we publish. This form provides structure for consistency and transparency in reporting. For further information on Nature Portfolio policies, see our Editorial Policies and the Editorial Policy Checklist.

Statistics

For all statistical analyses, confirm that the following items are present in the figure legend, table legend, main text, or Methods section.

n/a	Confirmed	
☐	☒	The exact sample size (*n*) for each experimental group/condition, given as a discrete number and unit of measurement
☐	☒	A statement on whether measurements were taken from distinct samples or whether the same sample was measured repeatedly
☐	☒	The statistical test(s) used AND whether they are one- or two-sided<br>*Only common tests should be described solely by name; describe more complex techniques in the Methods section.*
☐	☒	A description of all covariates tested
☐	☒	A description of any assumptions or corrections, such as tests of normality and adjustment for multiple comparisons
☐	☒	A full description of the statistical parameters including central tendency (e.g. means) or other basic estimates (e.g. regression coefficient) AND variation (e.g. standard deviation) or associated estimates of uncertainty (e.g. confidence intervals)
☐	☒	For null hypothesis testing, the test statistic (e.g. *F*, *t*, *r*) with confidence intervals, effect sizes, degrees of freedom and *P* value noted<br>*Give P values as exact values whenever suitable.*
☒	☐	For Bayesian analysis, information on the choice of priors and Markov chain Monte Carlo settings
☒	☐	For hierarchical and complex designs, identification of the appropriate level for tests and full reporting of outcomes
☐	☒	Estimates of effect sizes (e.g. Cohen's *d*, Pearson's *r*), indicating how they were calculated

Our web collection on statistics for biologists contains articles on many of the points above.

Software and code

Policy information about availability of computer code

Data collection	-
Data analysis	All analyses were performed using R version 4.1.1, RStudio and the package metafor.

For manuscripts utilizing custom algorithms or software that are central to the research but not yet described in published literature, software must be made available to editors and reviewers. We strongly encourage code deposition in a community repository (e.g. GitHub). See the Nature Portfolio guidelines for submitting code & software for further information.

Data

Policy information about availability of data

All manuscripts must include a data availability statement. This statement should provide the following information, where applicable:

- Accession codes, unique identifiers, or web links for publicly available datasets
- A description of any restrictions on data availability
- For clinical datasets or third party data, please ensure that the statement adheres to our policy

All data analyzed in this meta-analysis is available upon reasonable request from the corresponding author.

Human research participants

Policy information about studies involving human research participants and Sex and Gender in Research.

Reporting on sex and gender	Gender was considered as a moderating factor in our analysis. For each study we extracted the number of participants of each gender or the ratio of male and female study participants.
Population characteristics	All study participants were diagnose with a depressive disorder.
Recruitment	There were no participants recruited for this study. The data which was analyzed was extracted from published papers.
Ethics oversight	Approval from the local ethics committee was waived as no original data was acquired in the context of this study

Note that full information on the approval of the study protocol must also be provided in the manuscript.

Field-specific reporting

Please select the one below that is the best fit for your research. If you are not sure, read the appropriate sections before making your selection.

☒ Life sciences ☐ Behavioural & social sciences ☐ Ecological, evolutionary & environmental sciences

For a reference copy of the document with all sections, see nature.com/documents/nr-reporting-summary-flat.pdf

Life sciences study design

All studies must disclose on these points even when the disclosure is negative.

Sample size	No sample size calculation was performed.
Data exclusions	Studies were excluded if they: (1) included less than five participants, (2) included children or adolescents (<18 years), (3) focused exclusively on a more specific depression diagnosis (i.e. postpartum depression or late-life depression), or primarily investigated somatic (e.g. HIV, diabetes) or psychiatric main diagnose preceding depressive symptomatology (e.g. panic disorder), (4) employed a psychotherapeutic intervention based on psychoanalysis or culturally-adapted psychotherapy as well as therapy delivered by a telephone or group therapy of any therapy direction.<br>In case some relevant data was not reported in the published manuscripts of the studies identified during the literature search, we contacted authors via email in order to obtain the missing data. In some cases we did not receive any response or the needed data was not available. Studies were excluded from our meta-analysis, if data was not sufficient to calculate effect sizes as specified in the methods section.
Replication	Literature search and data extraction were conducted independently by two researchers (L.V. and UM.R.). Discrepancies were resolved in a consensus conference (L.K.I, L.V. and UM.R.). All information was checked for potential extraction errors independently by two researchers (N.D., J.W.).
Randomization	Randomization of original studies was analyzed in the assessment of study quality.
Blinding	Blinding of original studies was analyzed in the assessment of study quality.

Reporting for specific materials, systems and methods

We require information from authors about some types of materials, experimental systems and methods used in many studies. Here, indicate whether each material, system or method listed is relevant to your study. If you are not sure if a list item applies to your research, read the appropriate section before selecting a response.

Materials & experimental systems

n/a	Involved in the study	
☒	☐	Antibodies
☒	☐	Eukaryotic cell lines
☒	☐	Palaeontology and archaeology
☒	☐	Animals and other organisms
☐	☒	Clinical data
☒	☐	Dual use research of concern

Methods

n/a	Involved in the study	
☒	☐	ChIP-seq
☒	☐	Flow cytometry
☒	☐	MRI-based neuroimaging

Clinical data

Policy information about clinical studies

All manuscripts should comply with the ICMJE guidelines for publication of clinical research and a completed CONSORT checklist must be included with all submissions.

Clinical trial registration	https://osf.io/z45xr
Study protocol	https://osf.io/z45xr
Data collection	We conducted a systematic literature search in the PubMed database to identify all relevant studies published until January 11th, 2021.
Outcomes	Depressive and anxiety symptoms were assessed by self- or observer-rated clinical scales (e.g. Beck's Depression Inventory, Hamilton Depression Scale, State Trait Anxiety Inventory-STAI, Hamilton Anxiety Scale). In order to compare depressive symptom severity at baseline across studies, reported symptom measures were converted to BDI-II using published conversion procedures46,47. Psychosocial functioning was assessed using measures of global functioning (e.g. Global Assessment of Functioning), work-related functioning (e.g. Well-Being Inventory), social functioning (e.g. Social and Occupational Functioning Assessment Scale), health-related functioning (e.g. World Health Organization Quality of Life) and life quality (e.g. Quality of life scale). Adherence was quantified for all samples by the ratio of patients that did not drop out of the study and underwent an assessment after the intervention.
